# Potential protective effects of Azelnidipine against cerebral ischemia-reperfusion injury in male rats

**DOI:** 10.25122/jml-2022-0195

**Published:** 2022-11

**Authors:** Zainab Fakharaldeen, Ahmed Al-Mudhafar, Ali Radhi, Najah Hadi

**Affiliations:** 1 Department of Pharmacology and Therapeutics, Faculty of Medicine, University of Kufa, Kufa, Iraq; 2 Department of Medicine, Al-Hakeem Hospital, Al-Najaf Al-Ashraf, Iraq

**Keywords:** Azelnidipine, CI/RI, IL-6, IL-10, TNF-α, ICAM-1, NF-κB p65, BCCAO – Bilateral common carotid artery occlusion, CI/RI – Cerebral ischemia/reperfusion injury, CMC – Carboxymethyl cellulose, IHC – Immunohistochemistry, IL-6 – Interleukin-6, IL-10 – Interleukin-10, ICAM-1 – Intercellular adhesion molecule-1, NF-κB – Nuclear factor kappa B, PBS – Phosphate-buffered saline, T-AOC – Total anti-oxidant capacity, TNF-α – Tumor necrosis factor-α, TTC – 2,3,5-Triphenyltetrazolium chloride

## Abstract

This study was performed to evaluate the neuroprotective effect of Azelnidipine in cerebral ischemia/reperfusion and to envisage its mechanisms. Twenty-eight adult male Sprague-Dawley rats weighing 200–300 g were randomized into 4 groups (7 rats in each group). Sham (neck dissection without bilateral common carotid artery occlusion), control (30 minutes of bilateral common carotid artery occlusion and reperfusion for 1 hour), vehicle (identical volume of 0.3% carboxymethylcellulose (CMC) orally every day then bilateral common artery occlusion and reperfusion), and Azelnipine-treated rats (7 days of Azelnidipine pretreatment 3 mg/kg/day followed by bilateral common carotid artery occlusion and reperfusion). In addition to brain infarct volume and histopathological assessment, the brain tissues were harvested to evaluate cerebral IL-6, IL-10, TNF-α, ICAM-1, NF-κB p65, and total antioxidant capacity levels. Cerebral levels of IL-6, IL-10, TNF-α, NF-κB p65, and ICAM-1, besides cerebral infarct volume, were significantly elevated in control and vehicle related to sham groups, while total antioxidant capacity was markedly reduced. Azelnidipine treatment resulted in remarkable upregulation of total antioxidant capacity; meanwhile, IL-6, TNF-α, NF-κB p65, and ICAM-1 showed a considerable reduction. Cerebral IL-10 levels were not affected by Azelnidipine pretreatment. Histologically, control and vehicle rats showed severe ischemic injury, which was greatly reversed by Azelnidipine treatment. The current study disclosed that Azelnidipine could markedly reduce cerebral infarct volume and ameliorate histopathological damage in male rats exposed to cerebral ischemia/reperfusion. The neuroprotective effects of Azelnidipine probably stemmed from its anti-inflammatory and antioxidative properties. Azelnidipine had no effect on cerebral IL-10 levels.

## INTRODUCTION

Stroke is the second leading cause of death and disability globally, accounting for 5.7% of all disabilities and 11.6% of all deaths. 85% to 90% of all strokes are ischemic in nature, while intracerebral and subarachnoid hemorrhages account for 8.3% and 5.4%, respectively, of all strokes. The term “CI/RI” describes a time when the brain's blood flow is compromised, followed by a period when cerebral perfusion is restored. Despite the positive effects of reperfusion-restored cerebral blood flow, it can also have negative repercussions [1]. Reperfusion may result in a greater infarction than the initial occlusion and further deterioration of the nervous system. Additionally, the return of cerebral blood flow causes a hyperperfusion state that can result in brain edema and the hemorrhagic transition of an ischemic stroke. In cerebral ischemia with reperfusion, the blood-brain barrier is damaged more frequently (45%) than in individuals without reperfusion (18%) [2]. A fulminant invasion of leukocytes, which releases proinflammatory mediators and reactive oxygen species into the existing ischemic tissue, is the main cause of this reperfusion injury. This injury has a complex and multifactorial process. Additionally, platelet and complement activation, as well as mitochondrial damage, are the causes [3]. The brain has a high vulnerability to ischemia due to its excessive metabolic rate and its restricted ability to store glucose. When blood perfusion to a brain territory is reduced, the survival of that at-risk tissue depends upon the period and the severity of ischemia, tolerance of a particular brain region to ischemia, and the presence of collateral blood flow. The most susceptible brain cells to ischemia are neurons, followed by oligodendrocytes, astrocytes, and vascular cells. Specific brain areas have an extreme vulnerability to ischemia, like the hippocampal C1 region (3–5 minutes), pyramidal cells of cerebellar Purkinje cells, and the arterial border zones (watershed areas), whereas the medium-sized neurons of the striatum are more resistant (15–20 minutes) [4]. Penumbra is the reversibly damaged brain tissue around the ischemic core. The aim of stroke treatment is to rescue as large of the ischemic penumbra as promptly as possible. It has been described that about half of the patients with acute ischemic stroke yet have viable penumbras on magnetic resonance imaging (MRI) [5].

Azelnidipine is a third-generation dihydropyridine class antihypertensive drug that blocks voltage-gated (selectively L-type) calcium channels. Azelnidipine has been confirmed to have a neuroprotective effect against cerebral ischemia due to its high vascular selectivity, high lipophilic property, anti-inflammatory, antioxidative, antiapoptotic, anti-atherosclerosis properties, inhibition of catecholamine, and improvement of vascular endothelial function through increased nitric oxide activity [6]. In addition, dihydropyridine calcium channel blockers improve lipid metabolism, reduce the proliferation and migration of smooth muscle cells, and have antithrombotic and antiplatelet effects [7]. Azelnidipine also has anti-atherosclerotic action [8]. The blood flow to the brain was significantly increased in animal models treated with Azelnidipine [9]. Although Azelnidipine has potent anti-inflammatory and antioxidant properties, its neuroprotective effects against CI/RI are not fully studied. This study was designed to evaluate this aspect.

## MATERIAL AND METHODS

The study was carried out in the Department of Pharmacology/Faculty of Medicine, Kufa University. A total of 28 adult male Sprague-Dawley rats weighing 200–300 g were purchased from the animal house at Kufa Faculty of Science and housed in the same location in a temperature controlled (25±1℃) room with 60–65% humidity. Lights were maintained on a 12-hour light/dark cycle. The rats were allowed free food and water. Rats were randomly divided into 4 groups, each with 7 rats. Rats in Group 1 (Sham group) underwent identical surgical procedures without BCCAO. Rats in Group 2 (Control group) were subjected to BCCAO for 30 minutes and 1 hour of reperfusion without medication. Group 3 (Vehicle group): rats received an oral solution containing 0.3% CMC every day for seven days prior to surgery; after that, BCCAO for 30 minutes, followed by 1 hour of reperfusion. Rats in Group 4 (Azelnidipine-treated group) received 3 mg/kg/day of single dose Azelnidipine by gastric gavage for 7 days prior to surgery, followed by 30 minutes of BCCAO and then 1 hour of reperfusion.

### Induction of global cerebral ischemia

The induction of global cerebral ischemia was induced by BCCAO under general anesthesia with ketamine 100 mg/kg and xylazine 10 mg/kg intraperitoneally. Through a median neck incision, both common carotid arteries were exposed and occluded for 30 minutes to induce ischemia. The clamps were removed, and reperfusion was allowed for 1 hour.

### Sample preparation

The rats were decapitated, and the brains were isolated and kept on ice as quickly as possible and sectioned coronally for ELISA, histopathology, immunohistochemistry (IHC), and triphenyl tetrazolium chloride (TTC) stain.

### Preparation of brain tissue samples for ELISA

The homogenization was done by adding brain tissue, in a ratio of 1:10 (w/v), to a mixture of ice-cold 0.1M PBS containing 1x protease inhibitor cocktail and 0.2% Triton X-100 [10]. The brain tissues were homogenized by a sonicator, then centrifuged at 14000xg for 20 minutes at 4℃. The supernatants were aspirated, and the values of IL-6, IL-10, TNF-α, ICAM-1, and T-AOC were measured according to ELISA kit protocols.

### Preparation of brain tissue samples for histopathology

The brain sections were fixed in 10% formalin, engaged with an automated tissue processor, and stained with hematoxylin and eosin. The changes were scored as follows [11]:

0. (Normal): no damage;

1. (Mild): slight interstitial edema or Eosinophilic neurons;

2. (Moderate): at least two small hemorrhages;

3. (Severe): local necrosis.

*Cerebral infarct volume* was measured by immersion method with TTC stain [10].

*Cerebral levels of NF-κB* were assessed using the Dako Envision IHC technique.

### Preparation of Azelnidipine

The doses of Azelnidipine (purchased from ChemScene/USA, CAS No. 123524-52-7) were prepared immediately before use by suspending in 0.3% CMC and were given through oral gavage in a dose of 3 mg/kg/day [12, 13]. A low, non-hypotensive dose of Azelnidipine was used to document that the blood pressure-lowering mechanism is not involved in brain protection.

### Statistical analysis

Data were analyzed by employing SPSS version 26. Parametric data were assessed using the ANOVA test followed by the LSD post hoc test. Non-parametric test using Kruskal-Wallis test followed by post hoc test. P-value<0.05 was considered to be statistically significant.

## RESULTS

### Effects of Azelnidipine on cerebral IL-6, IL-10, TNF-α, ICAM-1, and T-AOC measured by ELISA

**Figure 1:**
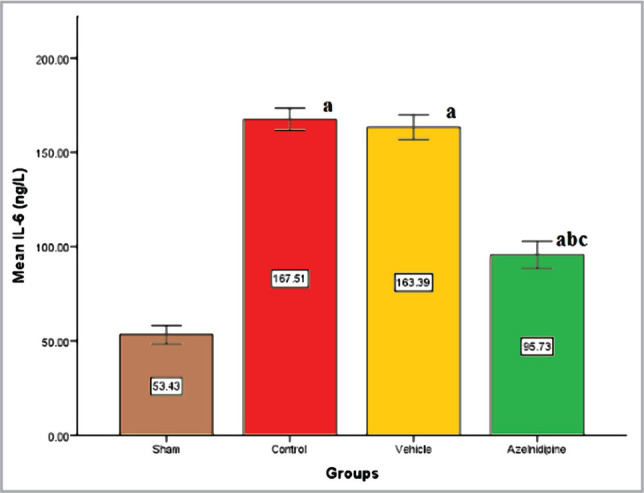
The mean of cerebral IL-6 (ng/L) of the four groups (n=7), value considers significant difference at p<0.05 when compared to; (a) sham group, (b) control group, (c) vehicle group.

**Figure 2:**
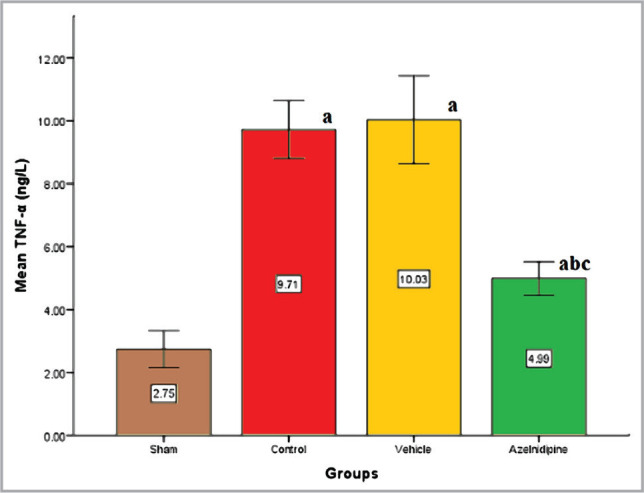
The mean of cerebral TNF-α (ng/L) of the four groups (n=7), value considers significant difference at p<0.05 when compared to; (a) sham group, (b) control group, (c) vehicle group.

**Figure 3:**
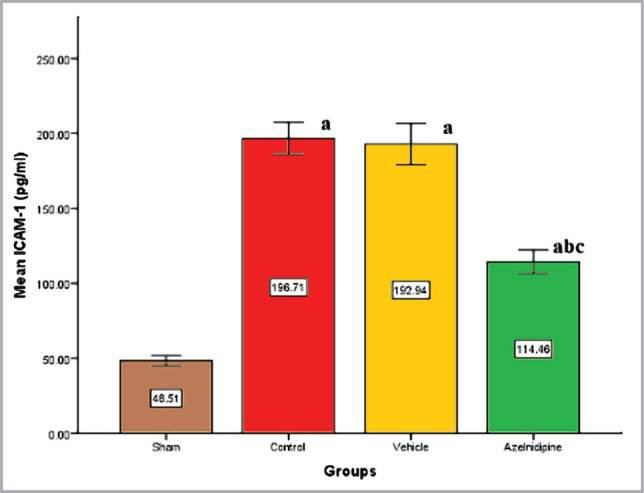
The mean of cerebral ICAM-1 (pg/ml) of the four groups (n=7), value considers significant difference at p<0.05 when compared to; (a) sham group, (b) control group, (c) vehicle group.

We found that the control and vehicle groups had markedly higher (p<0.05) IL-6, TNF-α, and ICAM-1 levels compared to the sham group, where these elevations were significantly reduced (p<0.05) in Azelnidipine-treated rats (Figures 1–3).

**Figure 4:**
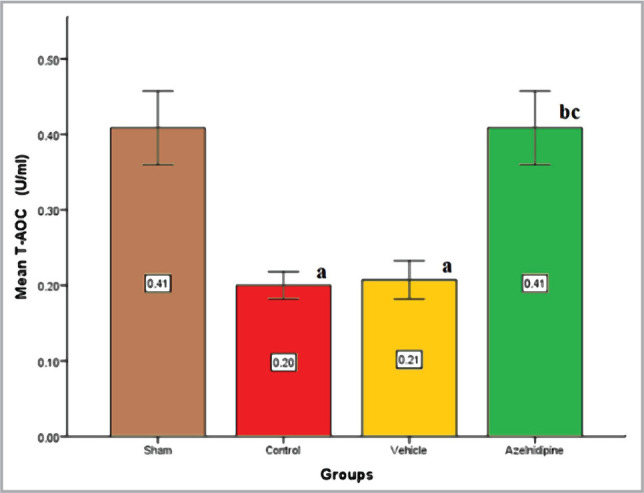
The mean of cerebral T-AOC (U/ml) of the four groups (n=7), value considers significant difference at p<0.05 when compared to; (a) sham group, (b) control group, (c) vehicle group.

Cerebral T-AOC levels were considerably reduced (p<0.05) in control and vehicle groups compared to sham rats. Compared to control and vehicle groups, these levels were significantly upregulated (p<0.05) by Azelnidipine treatment (Figure 4).

**Figure 5:**
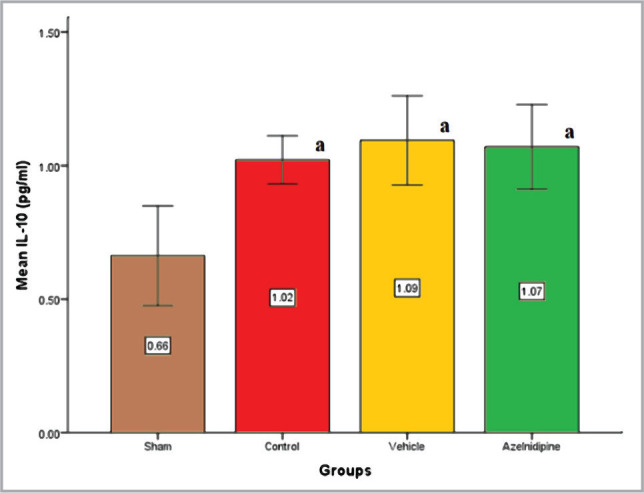
The mean of cerebral IL-10 (pg/ml) of the four groups (n=7), value considers significant difference at p<0.05; when compared to (a) sham group.

Compared to the sham rats, cerebral IL10 levels were dramatically increased (p<0.05) in the control and vehicle groups. These elevated levels were not changed by Azelnidipine treatment (Figure 5).

### Effects of Azelnidipine on cerebral NF-κB p65 measured by IHC

**Figure 6:**
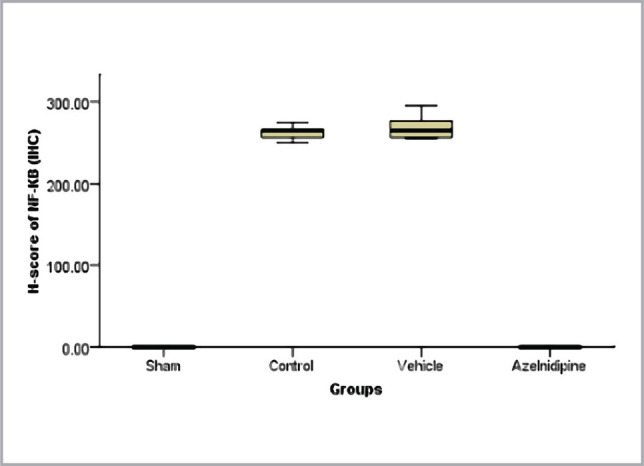
Kruskal Wallis box plot showing medians of cerebral NF-κB expression among the four groups, a significant difference (p<0.05) between sham vs. control and vehicle groups; control and vehicle vs. Azelnidipine-treated groups.

**Figure 7:**
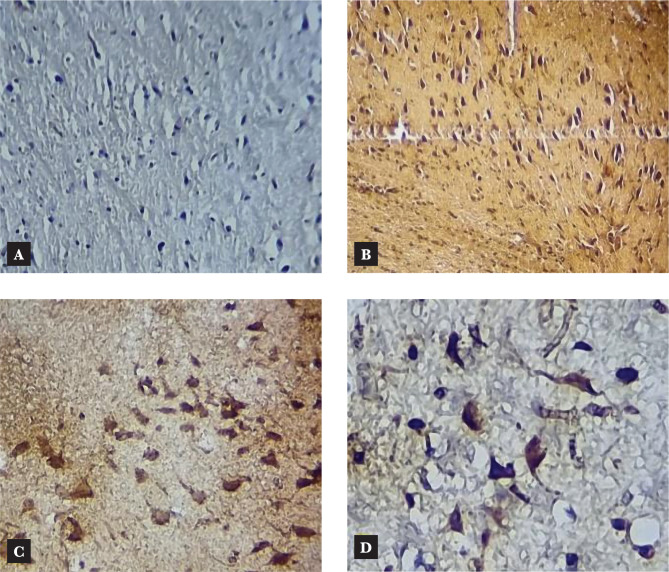
IHC of NF-κB nuclear expression in rat brain; A – Sham group: negative expression (X100); B – Control group; C – Vehicle group: intense nuclear expression (X200); D – Azelnidipine-treated group: weak expression (X200).

In our study, expression of NF-κB p65 was markedly higher (p<0.05) in control and vehicle rats compared to the sham group. This marked increment was reversed significantly (p<0.05) by Azelnidipine treatment (Figures 6 and 7 A–D).

### Effects of Azelnidipine on cerebral histopathology

**Figure 8:**
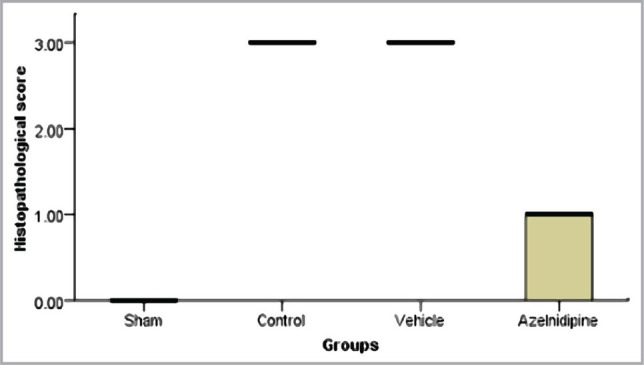
Kruskal Wallis box plot showing medians of histopathological ischemic changes among the four groups, significant difference (p<0.05) between sham vs. control and vehicle groups; Azelnidipine-treated vs. control and vehicle groups.

**Figure 9:**
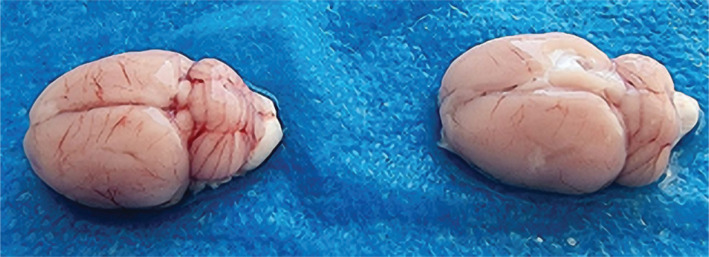
Gross pathology of rats' brains in CI/RI, (Left) Azelnidipine-treated group, (Right) Control group.

**Figure 10:**
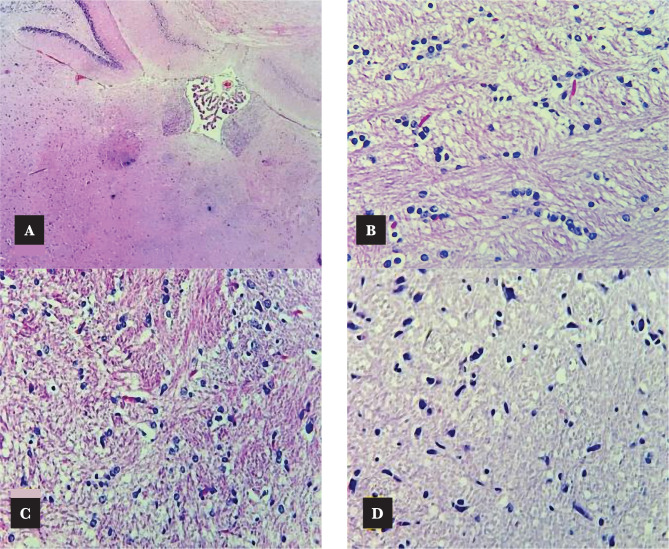
Cross section of rat cerebrum stained with hematoxylin and eosin; A – Sham group: normal cerebrum, cerebellum, and choroid plexus (X400); B – Control group; C – Vehicle group: severe interstitial edema with dark Eosinophilic neurons (X400); D – Azelnidipine-treated group: mild interstitial edema (X400).

The histopathological assessment showed a normal appearance in all sham rats. The control and vehicle groups showed remarkably severe p<0.05 ischemic damage. Azelnidipine pretreated rats expressed great amelioration p<0.05 in these changes (Figures 8,9 and 10 A–D).

### Effects of Azelnidipine on cerebral infarct size

**Figure 11:**
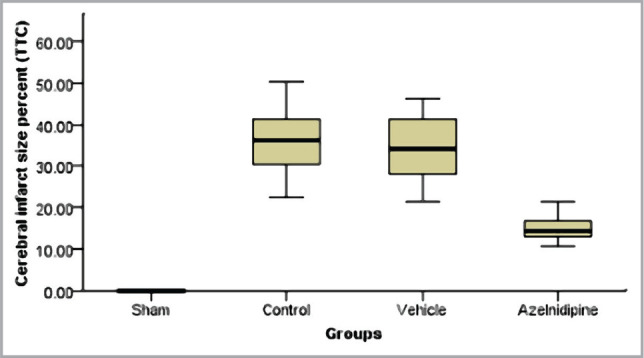
Kruskal Wallis box plot showing medians of cerebral infarct size among the four groups, significant difference p<0.05 between; sham vs. control and vehicle groups; Azelnidipine-treated vs. control and vehicle groups.

**Figure 12:**
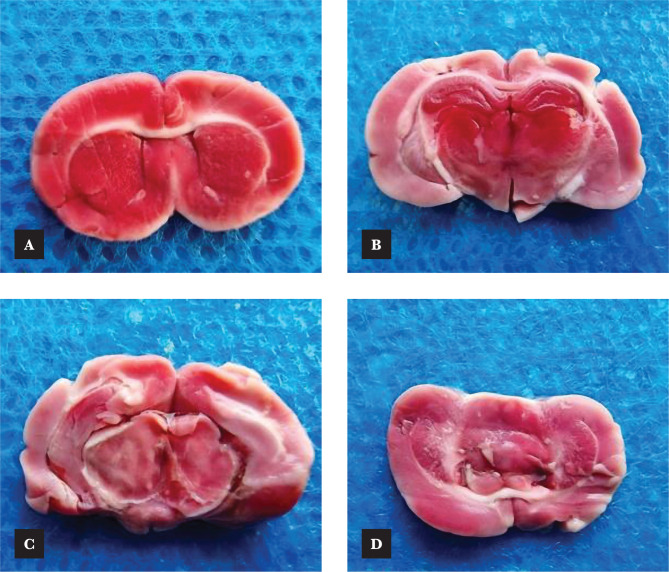
Photographs of coronal brain slices stained with TTC; A – Sham group; B – Control group; C – Vehicle group; D – Azelnidipine-treated group.

Our study showed a significant increment (p<0.05) in the infarction size percentage in control and vehicle compared to sham groups. This percentage was markedly ameliorated (p<0.05) in Azelnidipine-pretreated rats (Figures 11 and 12 A–D).

## DISCUSSION

Ischemic stroke is a devastating disease with high morbidity and mortality, affecting millions of people around the world. More than 700 drugs and extracts in preclinical animal studies have shown beneficial effects in CI/RI. However, currently, none of those drugs has been approved. Therefore, finding a novel therapeutic modality comprises a big challenge.

### Effects of Azelnidipine on cerebral IL-6, TNF-α, ICAM-1, and NF-κB p65

In our study, Azelnidipine pretreatment resulted in a significant downregulation (p<0.05) in the cerebral IL-6, TNF-α, ICAM-1 levels, and NF-κB p65 nuclear expression compared to the control and vehicle groups. These findings suggested that Azelnidipine has a significant anti-inflammatory effect on IL-6, TNF-α, ICAM-1, and NF-κB p65. Since nonhypotensive doses of Azelnidipine were used in the present study, this anti-inflammatory property was independent of the blood pressure-lowering effect of the drug. Normally, the proinflammatory cytokines and reactive oxygen species are found at low levels in the brain playing crucial roles in normal physiological functions. The CI/RI can result in strong inflammatory response that triggers the release of high levels of these factors, which can cause a greater cerebral injury than the initial ischemia. Interference with these pathways will greatly reduce the sequelae. Azelnidipine has strong anti-inflammatory properties that inhibit TNF, interleukins, and inflammatory cytokines [6]. The IL-6, TNF-α, ICAM-1, and NF-κB p65 play a key role in the inflammation. Azelnidipine suppression of these proinflammatory mediators will abolish a major portion of the inflammatory response. Subsequently, recruitment and adherence of circulating phagocytic cells (neutrophils and macrophage), stimulation of other immune cells to produce more proinflammatory mediators, oxidative stress, and increased blood-brain barrier permeability that triggered by IL-6, TNF-α, ICAM-1, and NF-κB p65 activation will be inhibited.

Furthermore, NF-κB stimulation can induce transcription of IL-1β and IL-8. The inhibition of NF-κB by Azelnidipine treatment will further restrict the inflammatory response. These effects explain why Azelnidipine has a strong anti-inflammatory effect. Ogawa et al. (2008) [14] demonstrated that serum TNF-α and IL-6 were greatly reduced in Azelnidipine-treated groups denoting that Azelnidipine is an efficient therapeutic option for inflammatory suppression. Kav et al. (2017) [15] showed that tissue levels of IL-6 and TNF-α were greatly decreased in the Azelnidipine-treated group of mice with dextran sulphate sodium (DSS)-induced colitis, suggesting Azelnidipine anti-inflammatory effect. To the best of our knowledge, there are no studies on the Azelnidipine effect on cerebral IL-6, TNF-α, ICAM-1 levels, and NF-κB p65 nuclear expression of rats exposed to CI/RI.

### Effects of Azelnidipine on cerebral IL-10 level

As a matter of interest, the cerebral IL-10 levels in our study displayed no significant difference between the Azelnidipine-treated group and the control and vehicle groups. These results suggested that the possible Azelnidipine neuroprotective effect was unrelated to IL-10. IL-10 is a strong anti-inflammatory cytokine. The cerebral IL-10 level in brain injury was significantly elevated to re-establish proinflammatory/anti-inflammatory homeostasis by inhibiting proinflammatory cytokines and reactive oxygen species production [16]. As far as we know, there are no studies on the Azelnidipine effect on cerebral IL-10 of rats.

### Effects of Azelnidipine on cerebral T-AOC level

In the current study, a significant increment (P<0.05) in the cerebral T-AOC was observed in the Azelnidipine-treated group compared to the control and vehicle groups. These results suggested that Azelnidipine has a neuroprotective effect through its antioxidant properties. Since nonhypotensive doses of Azelnidipine were used, this antioxidative property was independent of the hypotensive effect of the drug. T-AOC provides data on all integrated antioxidants present in the test solution, including enzymatic antioxidants like glutathione peroxidase, catalase, and superoxide dismutase, nonenzymatic antioxidants like glutathione and albumin, enzyme cofactors like coenzyme Q and lipoic acid, metabolites like uric acid and melatonin, and small molecule antioxidants like vitamin C and E and β–carotene. Lukic-Panin et al. (2007) [17], Nada et al. (2007) [7], Yamashita et al. (2009) [18], Omote et al. (2014) [19], and Gupta et al. (2020) [13] observed that Azelnidipine treatment in rats resulted in a great reduction of different markers of oxidative stress such as malondialdehyde (MDA) which is a product of lipid peroxidation. The antioxidant capacity of Azelnidipine is greater than that of other dihydropyridine calcium channel blockers [14, 20]. The anti-oxidative action of Azelnidipine may originate from its containment of an aromatic ring that can capture free radicals.

Nevertheless, the dihydropyridine ring in Azelnidipine is capable of donating proton that stabilizes free radicals [21]. Azelnidipine is lipophilic that simply passes through neuronal cells and counteracts lipid peroxidation [22]. Furthermore, Azelnidipine suppresses the intracellular influx of calcium, protecting neuronal cells from calcium overload, and creating free radicals [23]. Additionally, Azelnidipine can stimulate endothelial nitric oxide synthase (eNOS) and elevate nitric oxide (NO) synthesis. To the best of our knowledge, no studies have dealt with Azelnidipine effects on cerebral T-AOC assessment in rats.

### Effects of Azelnidipine on cerebral infarct size

The cerebral infarction size was significantly reduced (p<0.05) in the Azelnidipine-treated group in relation to the control and vehicle groups. Numerous studies indicate that stroke lesion size positively correlates with measured IL-6 and TNF-α levels [24, 25]. The results of our work are in line with that of Lukic-Panin et al. (2007) [17], Yamashita et al. (2009) [18], and Omote et al. (2014) [19]. Gupta et al. (2020) [13] supposed that Azelnidipine protection is due to its antioxidative and anti-inflammatory properties.

### Effects of Azelnidipine on cerebral histopathology

The azelnidipine-treated group displayed a significant amelioration (p<0.05) in the ischemic injury changes in relation to the control group. Azelnidipine-treated groups showed slight brain damage of mild interstitial edema and few dark Eosinophilic neurons. Our results are in accordance with that of Gupta et al. (2020) [13].

## CONCLUSION

Azelnidipine significantly ameliorated the cerebral infarct size, and the histopathological ischemic changes had neuroprotective effects in male rats exposed to CI/RI, probably through its anti-oxidative properties and also its anti-inflammatory effects on cerebral IL-6, TNF-α, ICAM-1, and NF-κB p65 nuclear expression. The neuroprotective effects of Azelnidipine were independent of its antihypertensive effect and unrelated to IL-10.
